# Supplementary figures and images for: Proximity labeling reveals a new in vivo network of interactors for the histone demethylase KDM5
Source: Epigenetics Chromatin. 2023 Feb 18;16:8. doi: 10.1186/s13072-023-00481-y (PMC9938590; doi:10.1186/s13072-023-00481-y)

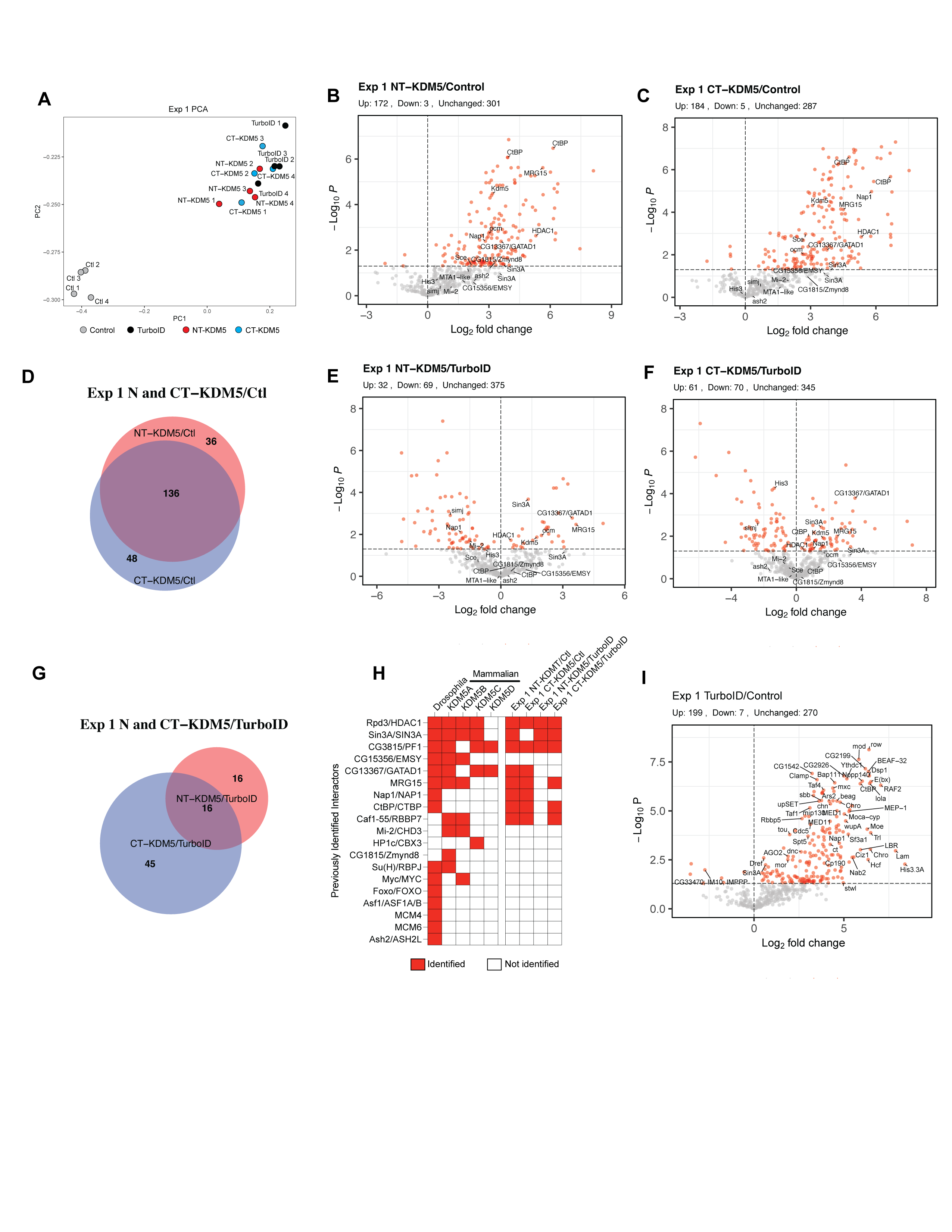

Supplement: Supplementary file 1 — Additional file 1. KDM5:TurboID experiment 1 using control and TurboID as controls. [file 13072_2023_481_MOESM1_ESM.tif]

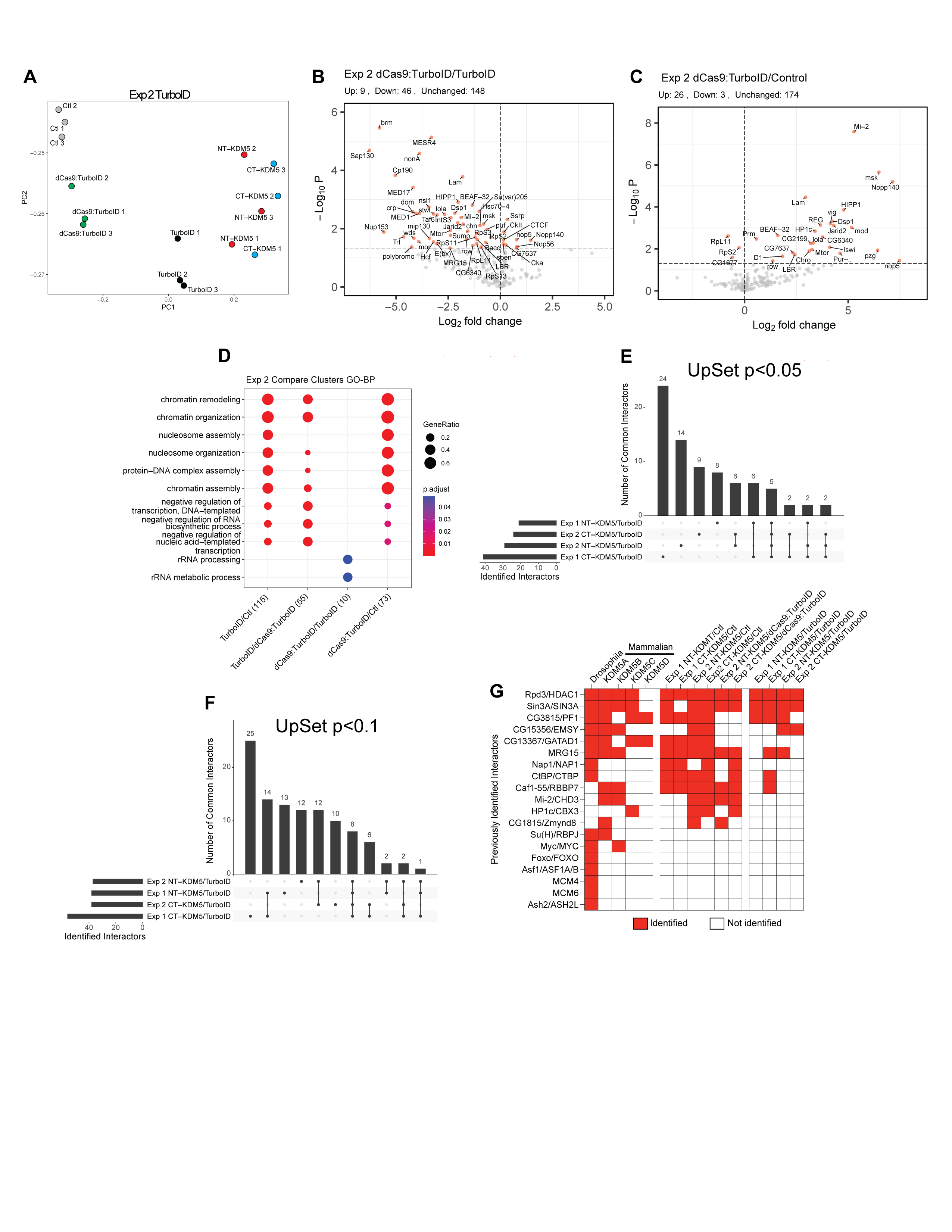

Supplement: Supplementary file 2 — Additional file 2. Analyses of KDM5:TurboID-enriched proteins using control, dCas9:TurboID and TurboID alone. [file 13072_2023_481_MOESM2_ESM.tif]

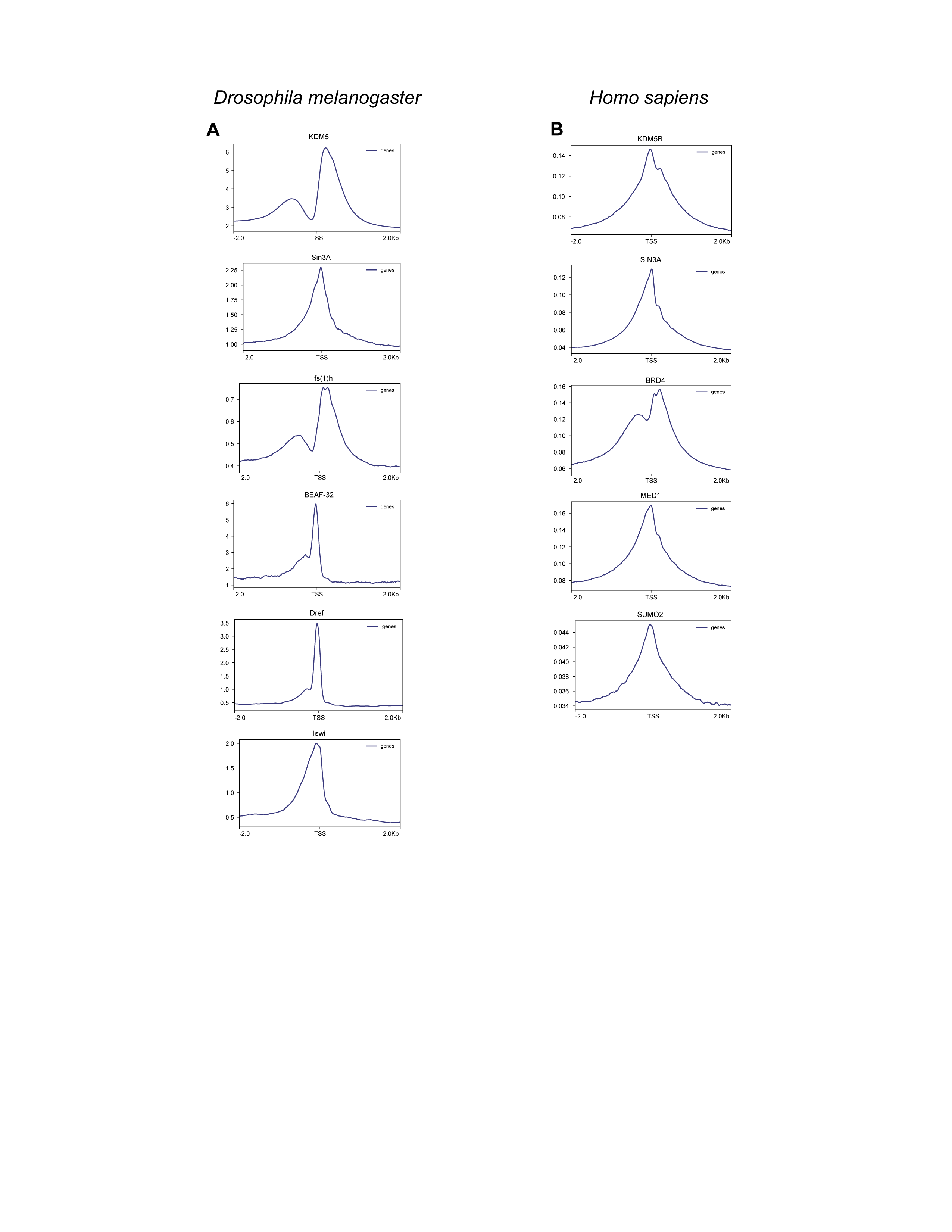

Supplement: Supplementary file 3 — Additional file 3. Binding profiles of KDM5 and identified interactors relative to the transcriptional start site. [file 13072_2023_481_MOESM3_ESM.tif]
