# Supplementary material for: Proximity labeling reveals a new in vivo network of interactors for the histone demethylase KDM5
Source: Epigenetics Chromatin. 2023 Feb 18;16:8. doi: 10.1186/s13072-023-00481-y (PMC9938590; doi:10.1186/s13072-023-00481-y)
Supplement: Supplementary file 5 — Additional file 5. Known interactors of Drosophila KDM5 and conservation of interaction in mammalian cells. [file 13072_2023_481_MOESM5_ESM.docx]

**Table S2 Known interactors of *Drosophila* KDM5 and conservation of interaction in mammalian cells**

| Complex | Protein | *Drosophila* | KDM5A | KDM5B | KDM5C | KDM5D | N- | C- |
| --- | --- | --- | --- | --- | --- | --- | --- | --- |
| HDAC1-related | HDAC1 | Gajan et al. ^1^  Lee et al. ^2^  Moshkin et al. ^3^ | Nishibuchi et al. ^4^  Varier et al. ^5^ | Klein et al. ^6^  Li et al. ^7^ | Tahiliani et al. ^8^ |  | Yes | Yes |
|  | Sin3A | Gajan et al. ^1^  Moshkin et al. ^3^  Spain et al. ^9^ | Ohguchi et al. ^10^  Varier et al. ^5^ |  |  |  | Yes | No |
|  | PF1 | Lee et al. ^2^  Moshkin et al. ^3^ | Nishibuchi et al. ^4^  Ohguchi et al. ^10^  Varier et al. ^5^ |  |  |  | Yes | Yes |
|  | EMSY | Moshkin et al. ^3^ | Nishibuchi et al. ^4^  Ohguchi et al. ^10^  Varier et al. ^5^ |  |  |  | Yes | Yes |
|  | GATAD1 | Lee et al. ^2^ | Ohguchi et al. ^10^  Varier et al. ^5^ |  |  |  | Yes | Yes |
| NuRD/NURF/HDAC1/Nap1 | Caf1 | - | Nishibuchi et al. ^4^  Varier et al. ^5^ | Klein et al. ^6^  Li et al. ^7^ |  |  | Yes | Yes |
| NuRD/Snf2/HDAC1 | Mi-2 | - | Nishibuchi et al. ^4^ | Klein et al. ^6^  Li et al. ^7^ |  |  | Yes | No |
|  | Zmynd8/CG1815 | - | Nishibuchi et al. ^4^  Ohguchi et al. ^10^ |  |  | Li et al. ^11^ | Yes | No |
| Tip60/Swr/HDAC1 | MRG15 | Lee et al ^2^  Moshkin et al. ^3^ | Varier et al. ^5^ |  |  |  | Yes | Yes |
| Chaperon/HDAC1 | NAP1 | Moshkin et al. ^3^ |  |  |  |  | Yes | No |
| ToRC | CtBP | Moshkin et al. ^3^ |  |  |  |  | Yes | Yes |
|  | Su(H) | Moshkin et al. ^3^ | Liefke et al. ^12^ |  |  |  | No | No |
|  | Myc | Secombe et al. ^13^ |  |  |  |  | No | No |
|  | Foxo | Li et al. ^14^ |  |  |  |  | No | No |
|  | Asf1 | Moshkin et al. ^3^ |  |  |  |  | No | No |
|  | MCM4 | Moshkin et al. ^3^ |  |  |  |  | No | No |
|  | MCM6 | Moshkin et al. ^3^ |  |  |  |  | No | No |
|  | Ash2 | Secombe et al. ^13^ |  |  |  |  | No | No |

**References:**

1 Gajan, A., Barnes, V. L., Liu, M., Saha, N. & Pile, L. A. The histone demethylase dKDM5/LID interacts with the SIN3 histone deacetylase complex and shares functional similarities with SIN3. *Epigenetics Chromatin* **9**, 4 (2016). <https://doi.org:10.1186/s13072-016-0053-9>

2 Lee, N., Erdjument-Bromage, H., Tempst, P., Jones, R. S. & Zhang, Y. The H3K4 Demethylase Lid Associates with and Inhibits the Histone Deacetylase Rpd3. *Mol Cell Biol* **29**, 1401-1410 (2009).

3 Moshkin, Y. M. *et al.* Histone chaperones ASF1 and NAP1 differentially modulate removal of active histone marks by LID-RPD3 complexes during NOTCH silencing. *Mol Cell* **35**, 782-793 (2009). <https://doi.org:S1097-2765(09)00516-4> [pii]

10.1016/j.molcel.2009.07.020

4 Nishibuchi, G. *et al.* Physical and functional interactions between the histone H3K4 demethylase KDM5A and the nucleosome remodeling and deacetylase (NuRD) complex. *J Biol Chem* **289**, 28956-28970 (2014). <https://doi.org:10.1074/jbc.M114.573725>

5 Varier, R. A. *et al.* Recruitment of the Mammalian Histone-modifying EMSY Complex to Target Genes Is Regulated by ZNF131. *J Biol Chem* **291**, 7313-7324 (2016). <https://doi.org:10.1074/jbc.M115.701227>

6 Klein, B. J. *et al.* The Histone-H3K4-Specific Demethylase KDM5B Binds to Its Substrate and Product through Distinct PHD Fingers. *Cell reports* (2014). <https://doi.org:10.1016/j.celrep.2013.12.021>

7 Li, Q. *et al.* Binding of the JmjC demethylase JARID1B to LSD1/NuRD suppresses angiogenesis and metastasis in breast cancer cells by repressing chemokine CCL14. *Cancer Res* **71**, 6899-6908 (2011). <https://doi.org:10.1158/0008-5472.CAN-11-1523>

8 Tahiliani, M. *et al.* The histone H3K4 demethylase SMCX links REST target genes to X-linked mental retardation. *Nature* **447**, 601-+ (2007).

9 Spain, M. M., Caruso, J. A., Swaminathan, A. & Pile, L. A. Drosophila SIN3 isoforms interact with distinct proteins and have unique biological functions. *J Biol Chem* **285**, 27457-27467 (2010). <https://doi.org:10.1074/jbc.M110.130245>

10 Ohguchi, H. *et al.* Lysine Demethylase 5A is Required for MYC Driven Transcription in Multiple Myeloma. *Blood Cancer Discov* **2**, 370-387 (2021). <https://doi.org:10.1158/2643-3230.BCD-20-0108>

11 Li, N. *et al.* ZMYND8 Reads the Dual Histone Mark H3K4me1-H3K14ac to Antagonize the Expression of Metastasis-Linked Genes. *Mol Cell* **63**, 470-484 (2016). <https://doi.org:10.1016/j.molcel.2016.06.035>

12 Liefke, R. *et al.* Histone demethylase KDM5A is an integral part of the core Notch-RBP-J repressor complex. *Genes Dev* **24**, 590-601 (2010). <https://doi.org:24/6/590> [pii]

10.1101/gad.563210

13 Secombe, J., Li, L., Carlos, L. S. & Eisenman, R. N. The Trithorax group protein Lid is a trimethyl histone H3K4 demethylase required for dMyc-induced cell growth. *Gene Dev* **21**, 537-551 (2007).

14 Liu, X., Greer, C. & Secombe, J. KDM5 interacts with Foxo to modulate cellular levels of oxidative stress. *PLoS Genet* **10**, e1004676 (2014). <https://doi.org:10.1371/journal.pgen.1004676>
